# Interim analysis of safety and efficacy of ruxolitinib in patients with myelofibrosis and low platelet counts

**DOI:** 10.1186/1756-8722-6-81

**Published:** 2013-10-29

**Authors:** Moshe Talpaz, Ronald Paquette, Lawrence Afrin, Solomon I Hamburg, Josef T Prchal, Katarzyna Jamieson, Howard R Terebelo, Gregory L Ortega, Roger M Lyons, Ramon V Tiu, Elliott F Winton, Kavita Natrajan, Olatoyosi Odenike, David Claxton, Wei Peng, Peter O’Neill, Susan Erickson-Viitanen, Lance Leopold, Victor Sandor, Richard S Levy, Hagop M Kantarjian, Srdan Verstovsek

**Affiliations:** 1 University of Michigan, Comprehensive Cancer Center, 1500 E Medical Center Dr, Ann Arbor MI 48109, USA; 2 UCLA Division of Hematology/Oncology, Los Angeles, CA, USA; 3 Medical University of South Carolina, Charleston, SC, USA; 4 Tower Cancer Research Foundation, Beverly Hills, CA, USA; 5 University of Utah School of Medicine, Salt Lake City, UT, USA; 6 University of Iowa College of Medicine, Iowa City, IA, USA; 7 Newland Medical Associates, Southfield, MI, USA; 8 Mid-Florida Hematology & Oncology Associates, Orange City, FL, USA; 9 Cancer Care Centers of South Texas/US Oncology, San Antonio, TX, USA; 10 Cleveland Clinic, Taussig Cancer Institute, Cleveland, OH, USA; 11 Emory University School of Medicine, Atlanta, GA, USA; 12 Georgia Regents University, Augusta, GA, USA; 13 University of Chicago, Chicago, IL, USA; 14 Penn State Hershey Cancer Institute, Hershey, PA, USA; 15 Incyte Corporation, Wilmington, DE, USA; 16 University of Texas MD Anderson Cancer Center, Houston, TX, USA

**Keywords:** Janus kinase inhibitor, Myelofibrosis, Phase II, Platelet count, Ruxolitinib, Spleen volume, Total symptom score

## Abstract

**Background:**

Ruxolitinib, a Janus kinase 1 and 2 inhibitor, demonstrated improvements in spleen volume, symptoms, and survival over placebo and best available therapy in intermediate-2 or high-risk myelofibrosis patients with baseline platelet counts ≥100 × 10^9^/L in phase III studies. The most common adverse events were dose-dependent anemia and thrombocytopenia, which were anticipated because thrombopoietin and erythropoietin signal through JAK2. These events were manageable, rarely leading to treatment discontinuation. Because approximately one-quarter of MF patients have platelet counts <100 × 10^9^/L consequent to their disease, ruxolitinib was evaluated in this subset of patients using lower initial doses. Interim results of a phase II study of ruxolitinib in myelofibrosis patients with baseline platelet counts of 50-100 × 10^9^/L are reported.

**Methods:**

Ruxolitinib was initiated at a dose of 5 mg twice daily (BID), and doses could be increased by 5 mg once daily every 4 weeks to 10 mg BID if platelet counts remained adequate. Additional dosage increases required evidence of suboptimal efficacy. Assessments included measurement of spleen volume by MRI, MF symptoms by MF Symptom Assessment Form v2.0 Total Symptom Score [TSS]), Patient Global Impression of Change (PGIC); EORTC QLQ-C30, and safety/tolerability.

**Results:**

By week 24, 62% of patients achieved stable doses ≥10 mg BID. Median reductions in spleen volume and TSS were 24.2% and 43.8%, respectively. Thrombocytopenia necessitating dose reductions and dose interruptions occurred in 12 and 8 patients, respectively, and occurred mainly in patients with baseline platelet counts ≤75 × 10^9^/L. Seven patients experienced platelet count increases ≥15 × 10^9^/L. Mean hemoglobin levels remained stable over the treatment period. Two patients discontinued for adverse events: 1 for grade 4 retroperitoneal hemorrhage secondary to multiple and suspected pre-existing renal artery aneurysms and 1 for grade 4 thrombocytopenia.

**Conclusions:**

Results suggest that a low starting dose of ruxolitinib with escalation to 10 mg BID may be appropriate in myelofibrosis patients with low platelet counts.

**Trial registration:**

ClinicalTrials.gov: NCT01348490.

## Background

Myelofibrosis (MF) is a Philadelphia chromosome-negative myeloproliferative neoplasm (MPN), including primary MF (PMF), post-polycythemia vera MF (PPV-MF) and post-essential thrombocythemia MF (PET-MF) [1]. MF is characterized by bone marrow fibrosis and extramedullary hematopoiesis, primarily in the spleen [2]. The clinical course of MF is varied, but it is associated with substantial morbidity and early mortality. Patients often develop debilitating constitutional and splenomegaly-related symptoms, which severely reduce quality of life (QoL) [1]. Hematologic manifestations include anemia, neutropenia and thrombocytopenia, with eventual progression to bone marrow failure and increased risk of acute myelogenous leukemia [1].

Dysregulated Janus kinase (JAK)-signal transducer and activator of transcription (STAT) signaling, as well as mutations in JAK2, are common in Philadelphia chromosome-negative MPNs [3]. The JAK-STAT pathway is essential for the regulation of myeloproliferation and immune response [4]. Ruxolitinib is a potent, orally administered inhibitor of JAK1 and JAK2 [5]. Ruxolitinib treatment reduced spleen volume and improved MF-related symptoms and QoL measures in patients with intermediate-2 or high-risk MF, as defined by the International Prognostic Scoring System (IPSS) [6], in the phase III COntrolled MyeloFibrosis Study with ORal JAK Inhibitor Treatment (COMFORT)-I and COMFORT-II studies [7,8]. Ruxolitinib was also associated with a survival advantage over placebo and best available therapy [7,9,10]. The most commonly observed adverse events (AEs) in the phase III trials were dose-dependent anemia and thrombocytopenia, which were anticipated as thrombopoietin and erythropoietin signal through JAK2 [11]. These events were manageable with dose interruption and titration, very rarely leading to treatment discontinuation. In addition to the efficacy and safety data from the COMFORT studies, exploratory analyses of bone marrow fibrosis samples from a phase I/II study [12] suggest that long-term treatment with ruxolitinib may delay the natural progression of bone marrow fibrosis seen in patients with myelofibrosis [13].

Among patients with PMF, approximately one-quarter have platelet counts <100 × 10^9^/L as a consequence of the disease [14-16]. Patients enrolled in the COMFORT trials, however, were required to have a baseline platelet count of ≥100 × 10^9^/L and received ruxolitinib starting doses of 15 or 20 mg twice daily. Therefore, a phase II study was conducted to assess the efficacy and safety of ruxolitinib when initiated at a lower starting dose (5 mg twice daily) with subsequent dose escalation in patients with MF who had baseline platelet counts of 50–100 × 10^9^/L. We present an interim analysis of 50 patients enrolled in this study.

## Methods

### Patients

Men or women ≥18 years of age with PMF, PPV-MF or PET-MF [17,18] were enrolled. Patients were required to have active symptoms, defined as one symptom score ≥5 or two symptom scores ≥3 at screening on the modified Myelofibrosis Symptom Assessment Form (MFSAF) version 2.0, which assessed night sweats, itching, abdominal discomfort, pain under ribs on left side, early satiety, bone/muscle pain and inactivity on a scale from 0 (absent) to 10 (worst imaginable) [7]. Eligible patients had platelet counts of 50–100 × 10^9^/L at screening and/or baseline visits, hemoglobin concentrations ≥65 g/L, peripheral blood blast count <5%, Dynamic International Prognostic Scoring System (DIPSS) [19] score ≥1, life expectancy 6 months or greater, Eastern Cooperative Oncology Group performance status ≤3, and were not being considered for stem cell transplant. Splenomegaly of any degree was not required for enrollment. Patients discontinued all MF treatments at least 14 days before the first dose of study medication.

Patients were excluded if they had well-controlled MF on current therapy; inadequate bone marrow reserve as demonstrated by absolute neutrophil count (ANC) <1.0 × 10^9^/L at screening visit, confirmed platelet count <50 × 10^9^/L, known history of platelet counts <25 × 10^9^/L in the absence of cytoreductive therapy or platelet transfusion(s) or ANC levels <500/μL in the 30 days before screening visit; major bleeding within 12 months of screening, requiring transfusion or resulting in hemoglobin decrease ≥30 g/L; history of esophageal/gastric varices or intracranial bleeding; or an international normalized ratio >1.5 times the upper limit of normal (ULN) or a partial thromboplastin time >1.5 times the ULN. Additional exclusion criteria were inadequate hepatic or renal function at screening and baseline visits as demonstrated by direct bilirubin ≥2 times the laboratory ULN, alanine aminotransferase >2.5 times the laboratory ULN, or creatinine >2.0 mg/dL; active bacterial, fungal, parasitic or viral infection; invasive malignancy in the previous 2 years; recent severe or unstable cardiac disease; splenic irradiation within 6 months; current therapy with moderate or potent cytochrome P450 3A4 inhibition; or previous JAK inhibitor therapy.

### Study design and treatment

This phase II, multicenter, open-label study is being conducted in the United States (ClinicalTrials.gov identifier: NCT01348490; study INCB018424-258). After a screening period of up to 21 days, eligible patients entered a 7-day baseline assessment phase followed by a 24-week treatment phase.

Ruxolitinib therapy was initiated at 5 mg twice a day. Optional dose increases were permitted beginning at week 4 in 5 mg once-daily increments every 4 weeks up to a dose of 10 mg twice daily if the following criteria were met: platelet counts remained ≥40 × 10^9^/L since the last scheduled study visit; the decline in platelet count, if decreased since the last study visit, was ≤20%; ANC was >1.0 × 10^9^/L since the last scheduled visit; no dose reductions or interruptions for safety occurred during the preceding 4-week interval; and any grade ≥2 hemorrhage was resolved. Dose increases beyond 10 mg twice daily, but not exceeding 15 mg twice daily, were permitted in patients who met these dose escalation criteria and, in addition, had inadequate response, defined as a Patient Global Impression of Change (PGIC) score of 3 (“minimally improved”) to 7 (“very much worse”). Dose increases after week 16 were not allowed unless the increase was related to recovery from a prior dose reduction or hold. Protocol-mandatory dose reductions were required for platelet counts ≥25 × 10^9^/L to <35 × 10^9^/L, and dose interruptions were required for platelet counts <25 × 10^9^/L, ANC <0.5 × 10^9^/L or grade ≥2 active hemorrhage. Dosing could be restarted or re-escalated when platelet counts recovered to ≥35 × 10^9^/L.

The study was approved by institutional review boards of participating institutions and was conducted in accordance with the Declaration of Helsinki, as outlined in the International Conference on Harmonization Guideline for Good Clinical Practice, and applicable regulatory requirements. All patients provided informed written consent.

### Endpoints and assessments

For this interim analysis, the following protocol-planned endpoints were evaluated: percentage change from baseline in spleen volume at week 24, as measured by magnetic resonance imaging (MRI) or computed tomography scan in patients who were not candidates for MRI or MRI was not available, and percentage change from baseline in Total Symptom Score (TSS) at week 24, as measured by the modified MFSAF version 2.0. MRI or CT scans were measured at baseline and at week 24 and read by a central reader blinded to initial treatment assignment. Spleen volume was calculated using a planimetry approach and validated software. Patients provided daily ratings for the severity of the following MF symptoms using the MFSAF version 2.0 electronic diary: night sweats, itching, abdominal discomfort, pain under ribs on left side, early satiety, bone/muscle pain and inactivity. Ratings for individual symptom severity ranged from 0 (absent) to 10 (worst imaginable). TSS is the sum of all individual symptoms with the exception of inactivity. Baseline TSS was the average of the daily scores for 7 days before initiation of study drug; week 24 TSS was the average of scores for the 28 days before the week 24 visit [7].

Additional protocol-planned endpoints in this interim analysis included the proportion of patients with a ≥35% reduction in spleen volume from baseline at week 24, the proportion of patients with a ≥10% reduction in spleen volume from baseline at week 24, the proportion of patients with a ≥50% improvement in TSS from baseline at week 24 and the percentage change in spleen length at each study visit. Spleen length below the left costal margin was measured by palpation at baseline and every 4 weeks. Exploratory endpoints included change from baseline in PGIC, assessed every 4 weeks, and the European Organization for Research and Treatment of Cancer Quality of Life Questionnaire-Core 30 (EORTC QLQ-C30), which was assessed at baseline and weeks 4, 12 and 24.

AEs were routinely monitored in all patients receiving at least one dose of ruxolitinib. All AEs were graded according to the National Cancer Institute’s Common Terminology Criteria for Adverse Events version 4.03.

### Analysis populations

As this is an ongoing study, not all patients were enrolled in the study for sufficient time to reach the week 24 visit. Therefore, changes from baseline in spleen volume, spleen length and TSS were based on patients with available data at week 24. Dose distribution and the responder analyses (proportion of patients achieving ≥35% reduction in spleen volume, ≥10% reduction in spleen volume or ≥50% reduction in TSS from baseline at week 24) were based on an intent-to-treat (ITT) population of patients who enrolled in the study at least 24 weeks before the data cutoff. This included patients who either completed the week 24 visit or discontinued from the study but would have reached the week 24 visit had they not discontinued from the study. For the dose distribution at week 24, patients with missing data were excluded from the analysis. For the responder analyses, patients who discontinued before week 24 and patients with missing values at week 24 were considered nonresponders; patients with missing baseline values were excluded. Safety analyses were based on all patients who received at least one dose of study drug. Additional file 1: Table S1 provides a detailed explanation of the number of evaluable patients for the dosing, efficacy (spleen volume, spleen length, and TSS) and safety analyses reported for this study.

## Results

### Patient characteristics and disposition

At the time of this interim analysis, a total of 50 patients had enrolled in this ongoing study. Baseline demographics and disease characteristics of the study population are shown in Table 1. Overall, the mean age was 69 years, 62% of patients had PMF, 62% were classified as intermediate-2 risk by the DIPSS and the mean platelet count was 72 × 10^9^/L. Of the 50 enrolled patients, 33 completed the week 24 visit, 8 discontinued before week 24 and 9 remained on study and have not yet completed their week 24 visit. Of the eight patients who discontinued, all were recruited at least 24 weeks before the data cutoff and were included in the ITT analyses of response. Primary reasons for discontinuation included AEs, withdrawal of consent and disease progression (n = 2 each), as well as death and other (n = 1 each).

**Table 1:** Patient demographics and disease characteristics at baseline

**Parameter**	**Value (N = 50)**
Mean age (range), years	69.3 (44–91)
Men,%	58
Race/ethnicity,%	
White	86.0
Black/African American	8.0
Asian	2.0
Pacific Islander	2.0
Other	2.0
Mean body mass index (SD), kg/m^2^	25.0 (4.37)
Myelofibrosis subtype,%	
PMF	62.0
PPV-MF	30.0
PET-MF	8.0
DIPSS risk category,%	
High	20.0
Intermediate-2	62.0
Intermediate-1	18.0
ECOG status,%	
0	12.0
1	74.0
2	12.0
Missing	2.0
History of transfusion in 12 weeks before baseline,%	40.0
Previous HU use,%	44.0
Mean platelet count (SD), × 10^9^/L	72.1 (21.9)
Mean hemoglobin (SD), g/L	97.3 (15.7)
Mean WBC (SD), × 10^9^/L	19.6 (20.2)
Mean TSS (SD)	19.4 (11.8)
Mean spleen length (SD), cm	13.4 (7.2)
Mean spleen volume (SD), cm^3^	2387.3 (1527.3)

Samples will be analyzed for JAK2V617F status at the end of study.

DIPSS, Dynamic International Prognostic Scoring System; ECOG, Eastern Cooperative Oncology Group; HU, hydroxyurea; JAK, Janus kinase; PET-MF, post-essential thrombocythemia myelofibrosis; PMF, primary myelofibrosis; PPV-MF, post-polycythemia vera myelofibrosis; SD, standard deviation; TSS, Total Symptom Score; WBC, white blood cell.

### Dosing and efficacy

**Figure 1:**
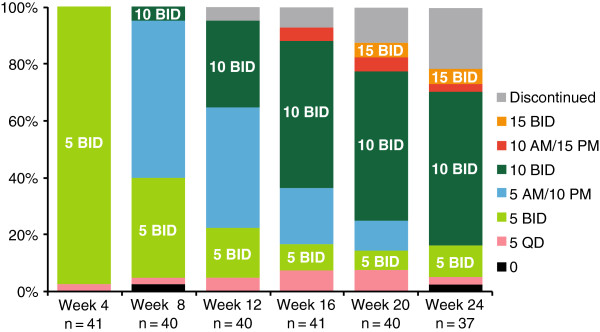
**Distribution of ruxolitinib daily dose over the 24-week study period.** N values represent patients with available dose information at the time of data analysis. Data shown for each time point represent the dose (in milligrams) that patients were receiving during the previous 4 weeks. BID, twice daily; QD, once daily.

Of the 41 patients who were evaluable for dosing, the median duration of exposure was 24.1 weeks. Dosing information was available for 37 patients at week 24. Most patients (23/37; 62%) were titrated to a ruxolitinib twice-daily dose of 10 mg or higher by week 24; 54% (20/37) of patients were receiving the 10 mg twice-daily dose at this time (Figure 1).

**Figure 2:**
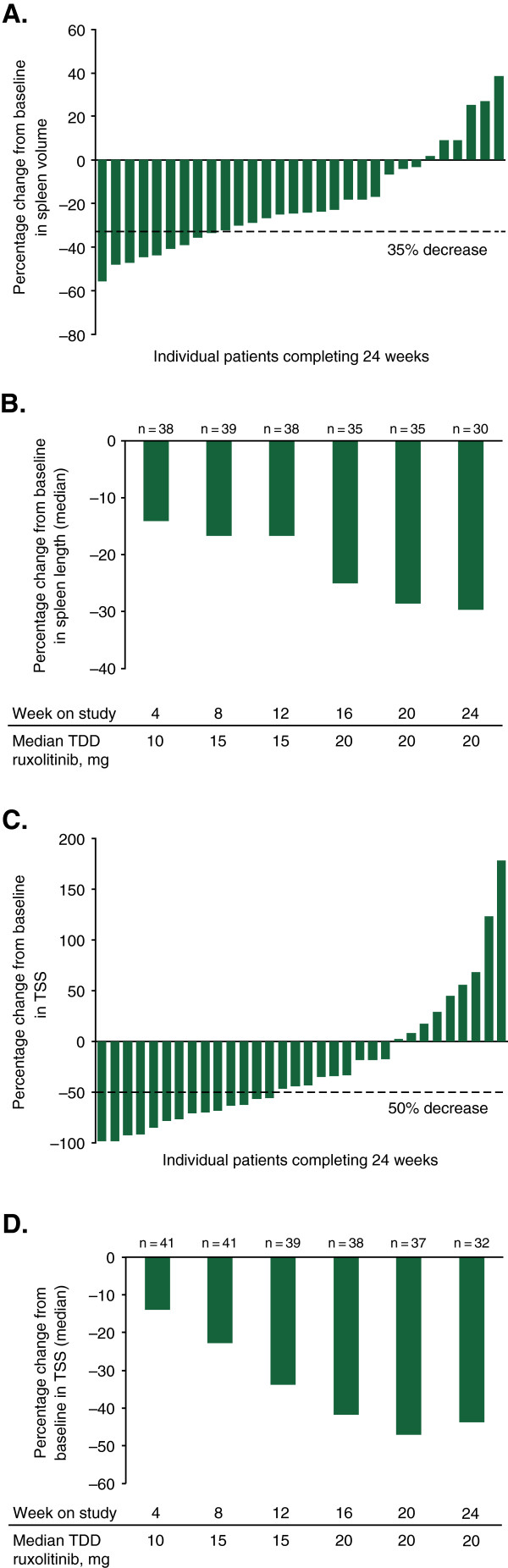
**Efficacy results at 24 weeks. (A)** Percentage change from baseline in spleen volume for individual patients at week 24. **(B)** Median percentage change from baseline in spleen length over time. **(C)** Percentage change from baseline in TSS in individual patients at week 24. **(D)** Median percentage change in TSS over time. Median dose is shown for patients with available dosing information. TDD, total daily dose; TSS, Total Symptom Score.

In patients who completed 24 weeks of therapy with available data (n = 30), clinically meaningful reductions from baseline in spleen volume and spleen length were observed at week 24. The median percentage change from baseline in spleen volume was –24.2% (range –55.8% to 38.5%). The majority of patients experienced some decrease in their spleen volume (Figure 2A). When evaluated by titrated dose (average dose over the last 4 weeks of the study, up to week 24), median percentage changes from baseline in spleen volume at week 24 were –16.7% for 5 mg once or twice daily (n = 7) and –28.5% for 10 mg twice daily (n = 20). By week 24, three patients had inadequate response and were escalated to doses >10 mg twice daily; the median percentage change from baseline in spleen volume in these patients was –6.6%. The median percentage change from baseline in spleen length at week 24 in the 30 patients with available data was –29.7% (range –100.0% to 58.3%). Improvements in spleen length were observed as early as week 4, and were maintained throughout the 24-week study period (Figure 2B). Forty patients were evaluable for the spleen volume responder analyses (Additional file 1: Table S1). A ≥35% reduction in spleen volume was experienced by eight patients (20.0%), and a ≥10% reduction occurred in 21 patients (52.5%). Notably, two patients who were considered nonresponders in the spleen volume responder analyses (because of missing MRI data at week 24) experienced clinically meaningful reductions in spleen length (–40.6% and –43.8%).

Decreases in TSS, indicating improvement in symptoms, were also observed in patients who completed 24 weeks of therapy (n = 32). The median percentage change from baseline in TSS at week 24 was –43.8% (range –98.6 to 178.6); most patients experienced some level of improvement in TSS (Figure 2C). Median percentage changes from baseline in TSS at week 24 by a titrated dose were 13.0% for 5 mg once or twice daily (n = 8) and –63.5% for 10 mg twice daily (n = 21). In the three patients who had to escalate to doses >10 mg twice a day because of inadequate response, median percentage change from baseline in TSS at week 24 was –33.8%. As observed with changes in spleen length, symptom improvements occurred at week 4 and were maintained throughout the 24-week study period (Figure 2D). In addition, median changes from baseline in the following individual symptoms of the modified MFSAF indicated improvement at week 24: abdominal discomfort (–40.0%), pain under left ribs (–50.0%), early satiety (–37.7%), night sweats (–73.0%), itching (–70.5%), bone or muscle pain (–37.3%) and inactivity (–28.8%). Forty-one patients were evaluable for the TSS responder analysis (Additional file 1: Table S1). At week 24, 14 patients (34.1%) experienced a ≥50% improvement in TSS.

**Figure 3:**
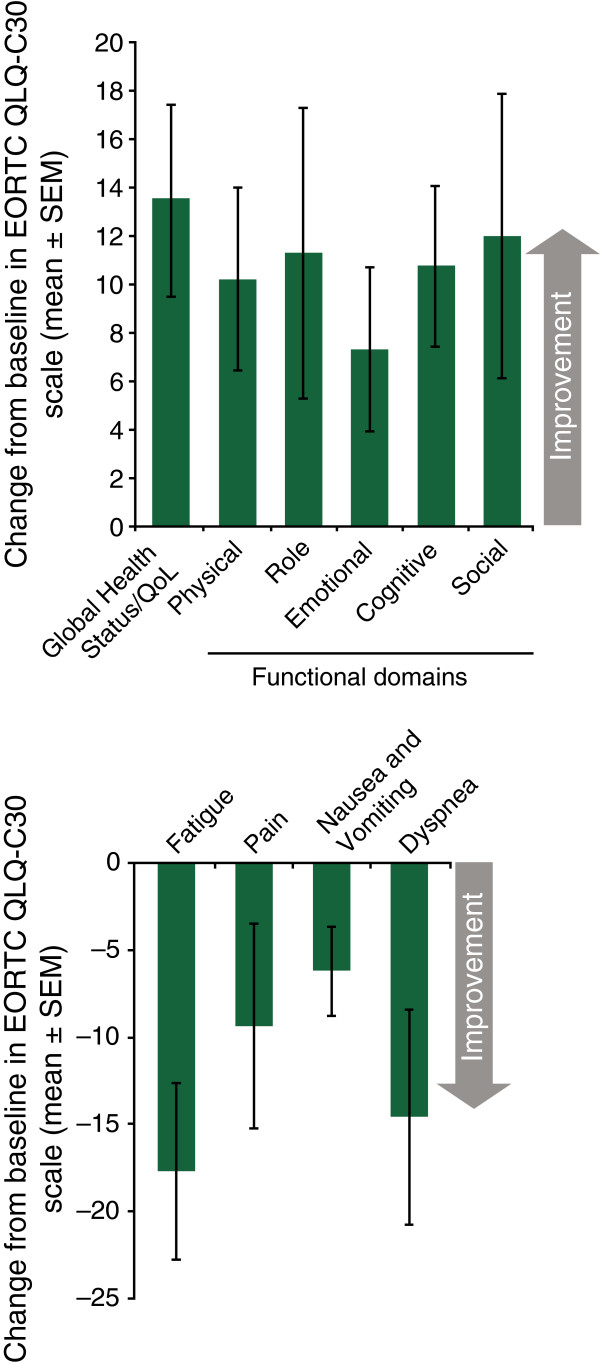
**Mean change from baseline to week 24 in quality of life, functional domains, and symptoms assessed by the EORTC QLQ-C30.***Note*: n = 32 for all scales except the Role Functioning and Cognitive Functioning domain, in which n = 31. EORTC QLQ-C30, European Organization for Research and Treatment of Cancer Quality of Life Questionnaire-Core 30; QoL, quality of life; SEM, standard error of the mean.

More than one-third of patients reported their symptoms as “much improved” or “very much improved,” as measured by the PGIC at week 4, which was before protocol-allowed dose optimization. By week 8 and continuing through week 24, more than one-half of the patients reported their symptoms to be at this level of improvement. Patients also reported improvements in QoL measures at week 24, including Global Health Status/QoL, as well as functional domains and most symptom scales of the EORTC QLQ-C30 (Figure 3).

### Safety and tolerability

A total of 50 patients received at least one dose of ruxolitinib and were included in the safety analyses. The most common nonhematologic AEs, regardless of causality, were diarrhea (28.0%), peripheral edema (26.0%), nausea (24.0%), abdominal pain (24.0%) and fatigue (22.0%) (Table 2). Grade 3 or 4 events of diarrhea, nausea, abdominal pain or fatigue each occurred in 4.0% of patients; no grade 3 or 4 events of peripheral edema were reported.

**Table 2:** Adverse events regardless of causality reported in the safety population (N = 50)

	**All Grades, n (%)**	**Grade 3 or 4, n (%)**
Nonhematologic AEs occurring in ≥10% of patients		
Diarrhea	14 (28.0)	2 (4.0)
Peripheral edema	13 (26.0)	0
Nausea	12 (24.0)	2 (4.0)
Abdominal pain	12 (24.0)	2 (4.0)
Fatigue	11 (22.0)	2 (4.0)
Upper respiratory tract infection	7 (14.0)	0
Vomiting	7 (14.0)	2 (4.0)
Hyperuricemia	6 (12.0)	2 (4.0)
Muscle spasm	6 (12.0)	0
Pyrexia	6 (12.0)	0
Constipation	5 (10.0)	0
Decreased appetite	5 (10.0)	0
Dizziness	5 (10.0)	0
Pleural effusion	5 (10.0)	0
New-onset hematologic AEs		
Hemorrhage	8 (16.0)*	1 (2.0)
Bruising (ecchymosis, contusion)	6 (12.0)	0
Laboratory values		
Anemia†	29 (64.4)	19 (42.2)‡
Thrombocytopenia	32 (64.0)	28 (56.0)§

*Grade ≥2 hemorrhage reported in four patients (8.0%).

†Denominator for percent calculation includes patients with grade 0, 1, or 2 anemia at baseline.

‡Grade 3 anemia events only are listed. According to CTCAE v4.03, grade 4 anemia requires a clinical assessment of “life-threatening consequences; urgent intervention indicated” and is not defined by a specific laboratory cutoff. One patient was reported to have experienced grade 4 anemia as an adverse event based on investigator clinical assessment.

§Grade 3 thrombocytopenia = 20 patients (40.0%); grade 4 thrombocytopenia = 8 patients (16.0%).

AE, adverse event; CTCAE, Common Terminology Criteria for Adverse Events.

Reductions in platelet counts to levels <35 and ≥25 × 10^9^/L required dose reductions per the study protocol and were experienced by 12 (24.0%) patients. Of these, nine had a baseline platelet count of ≤75 × 10^9^/L. Eight (16.0%) patients, seven of whom entered the study with a platelet count ≤75 × 10^9^/L at baseline, developed grade 4 thrombocytopenia (<25 × 10^9^/L). Of these patients, one patient with a baseline platelet count of 56 × 10^9^/L experienced grade 4 thrombocytopenia (platelet count of 19 × 10^9^/L) with grade 1 epistaxis after approximately 4 weeks of therapy. The patient had previously experienced grade 1 epistaxis during the screening period that resolved before the first dose of ruxolitinib. Dosing was interrupted and the patient received platelet transfusions. The thrombocytopenia did not resolve and the patient was discontinued from the study.

Events associated with bruising were reported in six (12.0%) patients, three (6.0%) patients reported ecchymosis and three (6.0%) patients reported contusion. All events were grade 1 with the exception of one event that was not graded at the time of the data cutoff. Grade 2 hemorrhage was reported in three (6.0%) patients: (i) epistaxis (5 mg AM/10 mg PM; event duration 1 day; no dose interruption); (ii) hematochezia concurrent with diverticulitis, abdominal pain and diarrhea (5 mg twice daily; event duration 18 days; no dose interruption); and (iii) rectal hemorrhage, resulting from exacerbation of internal hemorrhoids secondary to chronic constipation (5 mg twice daily; event duration 2 days; dose interrupted for 2 days). Before the bleeding events, platelet counts were >35 × 10^9^/L in each patient; all three remained on study and no platelet transfusions were required. One patient with suspected pre-existing renal aneurysms before study therapy initiation experienced grade 4 retroperitoneal hemorrhage secondary to multiple renal artery aneurysms, with acute renal failure resulting in discontinuation from the study. Before the event occurred, the patient’s platelet count was 71 × 10^9^/L.

**Figure 4:**
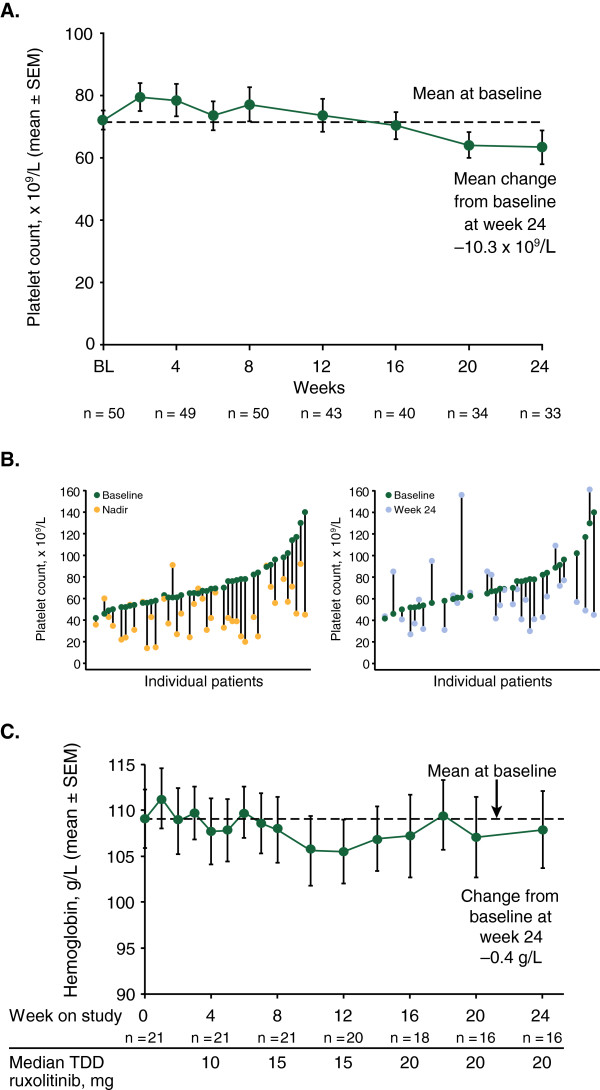
**Changes in hematologic parameters. (A)** Mean (SEM) platelet counts over time. **(B)** Changes in individual platelet counts from baseline to nadir (left panel) and baseline to week 24 (right panel). **(C)** Mean (SEM) hemoglobin levels over time in patients who did not receive red blood cell transfusions during the study. SEM, standard error of mean; TDD, total daily dose.

Although individual patients may have experienced platelet count reductions at various times during the study, the mean percentage change in platelet count for the study population was generally stable over the course of the 24-week treatment period (Figure 4A). Individual patients’ changes in platelet counts from baseline to nadir and baseline to week 24 (Figure 4B) showed that, with appropriate management, most patients were able to maintain adequate platelet counts during the course of the study. Seven patients had increases in platelet counts of ≥15 × 10^9^/L from baseline at week 24. These patients were generally younger, more recently diagnosed with MF, at lower risk by DIPSS, more likely to have PMF and had a lower neutrophil count at baseline compared with patients who had lesser increases or decreases in platelet counts (Additional file 2: Table S2).

Mean hemoglobin concentrations remained stable throughout the study. This was also observed in patients who did not receive transfusions (Figure 4C). The mean hemoglobin concentration in this subset of patients remained near the baseline level of 109 g/L throughout the study. Twenty patients required a red blood cell (RBC) transfusion in the 12 weeks before baseline. These patients, along with 10 out of 30 patients who did not require an RBC transfusion in the 12 weeks before baseline, required RBC transfusions during the treatment phase of the study.

The two patients who died during the study included a 67-year-old woman who had a fatal exacerbation of chronic obstructive pulmonary disease and a 68-year-old man who was reported to have died of natural causes.

## Discussion

This interim analysis from an ongoing phase II study suggests that a dosing strategy starting at 5 mg twice a day with gradual titration based on hematologic parameters and response can provide clinical benefit in patients with MF who have platelet counts of 50–100 × 10^9^/L. Reductions in palpable spleen length and improvements in symptoms were observed as early as week 4 when most patients were receiving ruxolitinib doses of 5 mg twice a day. By week 24, 62% of patients were able to achieve a stable ruxolitinib dose ≥10 mg twice daily, at which time the majority of patients had at least a 10% reduction in spleen volume, a response associated with clinically meaningful improvements in symptoms and QoL [20].

Reductions in spleen volume and improvements in TSS appeared to be greatest with the titrated dose of 10 mg twice daily. The small number of patients in the higher-titrated dose group did not experience the same level of efficacy; however, lack of response, as indicated by PGIC scores of 3 (“minimally improved”) to 7 (“very much worse”), was required for titration to doses >10 mg twice daily, confounding interpretation of a dose response at these doses.

In the phase III COMFORT-I study, which enrolled patients with platelet counts ≥100 × 10^9^/L, the median reductions in spleen volume and TSS at week 24 were 33.0% and 56.2%, respectively (versus 24.2% and 43.8%, respectively, in this analysis). Although patients in the COMFORT-I study started at higher doses (15 mg twice daily for patients with a baseline platelet count 100–200 × 10^9^/L and 20 mg twice daily for patients with a baseline platelet count >200 × 10^9^/L), the median titrated twice-daily doses at week 24 were 10 mg and 20 mg, respectively [21]. In a post hoc analysis of changes in spleen volume and TSS in COMFORT-I, patients with a final titrated dose of 10 mg twice daily achieved slightly lower spleen volume reductions and similar symptom score improvements as patients receiving higher ending doses [21]. Further, in the subgroup of patients in COMFORT-I who had baseline platelet counts 100–200 × 10^9^/L, the mean reduction in spleen volume was 23.6% and mean reduction in TSS was 33.4% at week 24 [21]. Our data suggest that patients with MF who have baseline platelet counts of 50–100 × 10^9^/L can initiate ruxolitinib and titrate to efficacious doses and experience clinically meaningful outcomes that compare with those seen in patients from COMFORT-I who had baseline platelet counts of 100–200 × 10^9^/L.

The most common nonhematologic AE was diarrhea, which was observed at a similar rate (28.0%) to that seen in patients from the COMFORT-I study receiving either ruxolitinib (23.2%) or placebo (21.2%) [7]. As expected, based on the mechanism of action of ruxolitinib and the lower starting platelet counts in this patient population, thrombocytopenia was the most common grade 3 or 4 AE. These events occurred mainly in patients with baseline platelet counts ≤75 × 10^9^/L and were managed with dose reductions or dose interruptions. Of interest, seven patients had increases in platelet counts of ≥15 × 10^9^/L. The characteristics of this small subgroup suggest that, patients who are younger and with less advanced MF may be at a lower risk for developing thrombocytopenia with ruxolitinib using the dosing scheme in this study.

Mean hemoglobin levels remained relatively stable over time, with a mean decrease of 0.4 g/L at week 24. Variation in mean hemoglobin during the current phase II study was similar to that observed in the placebo arm of the phase III COMFORT-I study [7]. In contrast, patients initiating ruxolitinib 15 or 20 mg twice daily in COMFORT-I experienced initial decreases in mean hemoglobin of approximately 10 g/L over the first 8 to 12 weeks that subsequently recovered to levels near baseline. Data from this phase II study are promising for patients at risk of cytopenias in that it shows use of a lower starting dose of ruxolitinib with gradual dose escalation did not result in a decrease in hemoglobin levels early in the course of ruxolitinib therapy, which was seen in COMFORT-I.

Preliminary findings from this study suggest that a dosing strategy starting with a lower dose of ruxolitinib with subsequent dose optimization can provide clinically meaningful reductions in spleen volume and TSS, and is generally well tolerated in patients with intermediate- or high-risk MF, as defined by DIPSS, who have platelet counts of 50–100 × 10^9^/L. Thrombocytopenia was manageable with dose reduction or dose interruption and mean hemoglobin levels remained stable throughout the study. Although this study is ongoing, data from this current analysis will help support individualized ruxolitinib dosing strategies in patients with MF who have lower platelet counts.

## Abbreviations

AE: Adverse event; ANC: Absolute neutrophil count; COMFORT: Controlled myelofibrosis study with oral JAK inhibitor treatment; DIPSS: Dynamic international prognostic scoring system; EORTC QLQ-C30: European organization for research and treatment of cancer quality of life questionnaire–core 30; IPSS: International prognostic scoring system; ITT: Intent-to-treat; JAK: Janus kinase; MF: Myelofibrosis; MPN: Myeloproliferative neoplasm; MRI: Magnetic resonance imaging; MSAF: Myelofibrosis symptom assessment form; PET-MF: Post-essential thrombocythemia myelofibrosis; PGIC: Patient global impression of change; PMF: Primary myelofibrosis; PPV-MF: Post-polycythemia vera myelofibrosis; QoL: Quality of life; RBC: Red blood cell; SD: Standard deviation; SEM: Standard error of the mean; STAT: Signal transducer and activator of transcription; TDD: Total daily dose; TSS: Total symptom score; ULN: Upper limit of normal; WBC: White blood cell.

## Competing interests

M.T. received research funding from Incyte Corporation and consultancy fees from Sanofi as a member of an advisory board; R.P. received consulting fees paid through his institution from Incyte Corporation; L.A., S.I.H., J.T.P., K.N., and D.C. have no relationships to disclose; H.R.T. received honoraria from Celgene, Millenium, and Amgen for speaking and consultancy fees for advisory board membership from Celgene; K.J. received membership on an entity’s Board of Directors or advisory committees from Sunesis and consultancy fees from Blue Distinction Centers for Transplants and BlueCross BlueShield Association; G.L.O. received research funding from Incyte Corporation; R.M.L. received grant support through his institution from Incyte Corporation; R.V.T., serves on speakers bureau from Incyte Corporation; E.F.W. received support through his institution from Incyte Corporation and Sanofi-Aventis; O.O. received grant support through her institution from Incyte Corporation, grant support for clinical trials from S*Bio, NS Pharma, Celgene, and Novartis, membership on an entity’s advisory committees from Incyte Corporation, Sanofi-Aventis, Spectrum Pharmaceuticals, and Sunesis, and honoraria for CME-related speaking engagements and research support from Topotarget and Eisai; W.P., P.O., L.L., V.S., and R.S.L. are employees of Incyte Corporation and own stock in Incyte Corporation; S.E.-V. is a former employee of Incyte Corporation and owns stock in Incyte Corporation; H.M.K. received grant support through his institution from Incyte Corporation; S.V. received grant support through his institution from Incyte Corporation, Exelixis, Celgene, NS Pharma, Infinity Pharmaceuticals, SBIO, Lilly Oncology, AstraZeneca, Geron, Bristol-Myers Squibb, YM BioSciences, Gilead, and Roche.

## Authors’ contributions

MT and SV performed the research and contributed to concept design and data collection. RP, LA, SIH, JTP, KJ, HRT, GLO, RML, RVT, EFW, KN, OO, DC, and HMK performed the research and contributed to data collection. WP performed statistical analyses and contributed to data interpretation. PO contributed to data collection. SE-V, LL, VS, and RSL contributed to concept design and data interpretation. All authors assisted with drafting the manuscript and/or critical revision of the content, and approved of the final manuscript submitted.

## Authors’ information

Susan Erickson-Viitanen is a former employee of Incyte Corporation.

## Supplementary Material

Additional file 1: Table S1 Explanation of patient populations analyzed for dosing, efficacy, and safety analyses. Click here for file

Additional file 2: Table S2 Baseline characteristics of patients with increase in platelet count ≥15 × 10^9^/L and those with increase in platelet count <15 × 10^9^/L or decreases in platelet count. Click here for file

## **Study investigators**

The following investigators contributed to the study (listed in alphabetical order): Lawrence Afrin, Medical University of South Carolina, Charleston, SC; Murat O. Arcasoy, Duke University Medical Center, Durham, NC; Maria Baer, University of Maryland Marlene and Stewart Greenebaum Cancer Center, Baltimore, MD; Thomas Carter, University of Iowa College of Medicine, Iowa City, IA; David Claxton, Penn State Hershey Cancer Institute, Hershey, PA; Solomon I. Hamburg, Tower Cancer Research Foundation, Beverly Hills, CA; Jimmie Harvey, Birmingham Hematology and Oncology Associates, LLC, Birmingham, AL; Chris Holmes, University of Vermont College of Medicine, Colchester, VT; Katarzyna Jamieson, University of Iowa College of Medicine, Iowa City, IA; Hagop M. Kantarjian, University of Texas MD Anderson Cancer Center, Houston, TX; Roger M. Lyons, Cancer Care Centers of South Texas/US Oncology, San Antonio, TX; Kavita Natrajan, Georgia Regents University, Augusta, GA; Olatoyosi Odenike, University of Chicago, Chicago, IL; Richard Orlowski, Carolina Oncology Specialists, PA, Hickory, NC; Gregory L. Ortega, Mid-Florida Hematology & Oncology Associates, Orange City, FL; Ronald Paquette, UCLA Division of Hematology/Oncology, Los Angeles, CA; Nikolai Podoltsev, Yale University School of Medicine, New Haven, CT; Josef T. Prchal, University of Utah School of Medicine, Salt Lake City, UT; Alfred Saleh, Sharp Clinical Oncology Research, San Diego, CA; Michael Savona, Sarah Cannon Research Institute, Sarah Cannon Center for Blood Cancers, Tennessee Oncology, Nashville, TN; Michael Scola, Hematology-Oncology Associates of Northern New Jersey, Morristown, NJ; Richard Silver, Weill Cornell Medical Center, New York, NY; Moshe Talpaz, University of Michigan, Ann Arbor, MI; Howard R. Terebelo, Newland Medical Associates, Southfield, MI; Ramon V. Tiu, Cleveland Clinic, Taussig Cancer Institute, Cleveland, OH; Srdan Verstovsek, University of Texas MD Anderson Cancer Center, Houston, TX; Elliott F. Winton, Emory University School of Medicine, Atlanta, GA; Steven Young, Somerset Hematology Oncology Associates, PA, Somerville, NJ.
