# Supplementary material for: Interim analysis of safety and efficacy of ruxolitinib in patients with myelofibrosis and low platelet counts
Source: J Hematol Oncol. 2013 Oct 29;6:81. doi: 10.1186/1756-8722-6-81 (PMC4176265; doi:10.1186/1756-8722-6-81)
Supplement: Additional file 1: Table S1 — Explanation of patient populations analyzed for dosing, efficacy, and safety analyses. [file 1756-8722-6-81-S1.pdf]

Additional Table 1:

Explanation of patient populations analyzed for dosing, efficacy, and safety analyses

		Patients, n				
**Analysis**	**Population**	**Completed wk 24**	**Discontinued before wk 24**	**Ongoing**	**Excluded or missing data**	**Evaluable**
**Dosing**, wk 24	ITT population (all pts enrolled 24 wks before data cutoff)	33	8	N/A	4 missing data	37
**Efficacy, from baseline at wk 24**						
Spleen						
% Change in spleen volume<br>% Change in spleen length	All pts who completed wk 24 with available data	33	N/A	N/A	3 missing data at wk 24[a]	30
Spleen volume response (≥10%, ≥35% reduction)	ITT population (all pts enrolled in study 24 wks before data cutoff)	33	8	N/A	1 excluded[b]	40
Total Symptom Score (TSS)						
% change in TSS	All pts who completed wk 24 with available data	33	N/A	N/A	1 missing data at wk 24	32
TSS response (≥50% reduction)	ITT population (all pts enrolled in study 24 wks before data cutoff)	33	8	N/A	0	41
**Safety**	All pts who received ≥1 dose of ruxolitinib	33	8	9	N/A	50

[a] 3 patients with missing data: (1) splenectomy before study entry; (2) refusal to undergo MRI; (3) week 24 MRI performed outside of window.
[b] 1 patient excluded for missing baseline value (splenectomy before study entry).
ITT population included all patients who completed the week 24 study visit and all patients who would have completed the week 24 study visit had they not discontinued from the study.
ITT, intent-to-treat; MRI, magnetic resonance imaging; N/A, not applicable; pts, patients.
