# Supplementary material for: Interim analysis of safety and efficacy of ruxolitinib in patients with myelofibrosis and low platelet counts
Source: J Hematol Oncol. 2013 Oct 29;6:81. doi: 10.1186/1756-8722-6-81 (PMC4176265; doi:10.1186/1756-8722-6-81)
Supplement: Additional file 2: Table S2 — Baseline characteristics of patients with increase in platelet count ≥15 × 109/L and those with increase in platelet count <15 × 109/L or decreases in platelet count. [file 1756-8722-6-81-S2.pdf]

Interim analysis of safety and efficacy of ruxolitinib in patients with myelofibrosis and low platelet counts

Talpaz M, et al.

Appendix:

Study INCB018424-258 Investigators

The following investigators contributed to the study (listed in alphabetical order):

Lawrence Afrin, Medical University of South Carolina, Charleston, SC; Murat O. Arcasoy, Duke University Medical Center, Durham, NC; Maria Baer, University of Maryland Marlene and Stewart Greenebaum Cancer Center, Baltimore, MD; Thomas Carter, University of Iowa College of Medicine, Iowa City, IA; David Claxton, Penn State Hershey Cancer Institute, Hershey, PA; Solomon I. Hamburg, Tower Cancer Research Foundation, Beverly Hills, CA; Jimmie Harvey, Birmingham Hematology and Oncology Associates, LLC, Birmingham, AL; Chris Holmes, University of Vermont College of Medicine, Colchester, VT; Katarzyna Jamieson, University of Iowa College of Medicine, Iowa City, IA; Hagop M. Kantarjian, University of Texas MD Anderson Cancer Center, Houston, TX; Roger M. Lyons, Cancer Care Centers of South Texas/US Oncology, San Antonio, TX; Kavita Natrajan, Georgia Regents University, Augusta, GA; Olatoyosi Odenike, University of Chicago, Chicago, IL; Richard Orlowski, Carolina Oncology Specialists, PA, Hickory, NC; Gregory L. Ortega, Mid-Florida Hematology & Oncology Associates, Orange City, FL; Ronald Paquette, UCLA Division of Hematology/Oncology, Los Angeles, CA; Nikolai Podoltsev, Yale University School of Medicine, New Haven, CT; Josef T. Prchal, University of Utah School of Medicine, Salt Lake City, UT; Alfred Saleh, Sharp Clinical Oncology Research, San Diego, CA; Michael Savona, Sarah Cannon Research Institute, Sarah Cannon Center for Blood Cancers, Tennessee Oncology, Nashville, TN; Michael Scola, Hematology-Oncology Associates of Northern New Jersey, Morristown, NJ; Richard Silver, Weill Cornell Medical Center, New York, NY; Moshe Talpaz, University of Michigan, Ann Arbor, MI; Howard R. Terebelo, Newland Medical Associates, Southfield, MI; Ramon V. Tiu, Cleveland Clinic, Taussig Cancer Institute, Cleveland, OH; Srdan Verstovsek, University of Texas MD Anderson Cancer Center, Houston, TX; Elliott F. Winton, Emory University School of Medicine, Atlanta, GA; Steven Young, Somerset Hematology Oncology Associates, PA, Somerville, NJ.
